# Cloacal malformation: A rare case report and review of prenatal imagings

**DOI:** 10.1097/MD.0000000000021839

**Published:** 2020-10-16

**Authors:** Ge Huang, Chang-Jun Zheng, Guang-Yu Chu, Shu-Yan Liu

**Affiliations:** a Department of Radiology; b Department of Orthopaedic; c Department of Gynaecology, the Second Hospital of Jilin University, Changchun, Jilin Province, China.

**Keywords:** cloaca, fetal, magnetic resonance imaging, malformation, prenatal, ultrasound

## Abstract

**Rationale:**

Cloacal malformation (CM) is a serious type of anorectal and urogenital tract malformation. However, prenatal ultrasound (US) detection of CM is challenging. In this paper, we reported a rare case of CM prenatally diagnosed by US and magnetic resonance imaging (MRI), as well as reviewed the prenatal US and MRI characteristics of CM in the literature.

**Patient concerns:**

A 30-year-old pregnant woman complained of cystic mass in the fetal abdomen detected by prenatal US.

**Diagnosis:**

Fetus CM.

**Interventions:**

The fetus was diagnosed as fetal CM by US and MRI, then the pregnant woman received a drug-induced labor treatment. After the neonate was delivered, the measurement was performed on the weight, length, head circumference, abdomen circumference, and bilateral thigh circumference.

**Outcomes:**

A female dead neonate was delivered from the vagina of the gravida, showing congenital anus absence. Prenatal ultrasound demonstrated right kidney duplication, hydronephrosis, and right ureteral dilatation. Meanwhile, prenatal MRI showed a cystic cavity, double collecting systems of right kidney, right ureteral dilatation, and right rectum dilatation. In addition, general parameters are as follows: weight: 2280 g; length: 39 cm; head circumference: 26.3 cm; abdomen circumference: 31 cm; right thigh circumference: 17 cm, and left thigh circumference: 18 cm.

**Lessons:**

US combined with MRI can not only provide reliable evidence for fetal CM in the third trimester but also offer crucial information to the pregnant women to establish clinic treatment programs as early as possible.

## 1 Introduction

Cloacal malformation (CM) is an extremely rare disease with an incidence of approximately 0.002%,^[1–7]^ which represents the most severe classification of urogenital and anorectal deformity.^[6]^ The CM patients are characterized by the convergence of the urinary, vagina, rectum, and tract into a single shared channel that is open at the urethral meatus.^[6–10]^

The prenatal diagnosis of CM is difficult due to the extraordinary lack of cases.^[8–11]^ Surgical intervention for the disease is usually decided within the first few hours after birth, so delaying the timing of surgery can seriously affect the quality of life of a newborn.^[6]^ Therefore, confident prenatal diagnosis is quite necessary. In the past, the ultrasound (US) was widely used to identify the large abdominal cystic mass, but not to ascertain in most situations.^[12]^ In recent years, authors have reported that fetal MRI might provide more definitive evidence.^[9,13–16]^

Therefore, pregnant women should receive regular prenatal US examinations. Once the abdominal mass is detected, further careful evaluation of MRI is necessary to help make a definite prenatal diagnosis or at least to suspect the fetus CM. To contribute to the available database on the fetus CM, we reported a rare case of CM prenatally diagnosed by US and MRI, described the features of images, and also reviewed the prenatal US and MRI characteristics of CM in literature.

## 2 Case report

### 2.1 Materials and methods

The study was approved by the ethics committee of the Second Hospital of Jilin University, and the informed consent was gained from the patient.

### 2.2 General characteristics of patients

A 30-year-old pregnant woman, gravida 2, para 0, with 33 weeks and 6 days of gestation, admitted to the outpatient department of gynecology and obstetrics. The prenatal US showed a cystic mass in the fetal abdomen. No obvious abnormality was found in non-invasive DNA and OGTT. No positive past medical history and family genetic history were observed. The gravida was no abdominal pain and specifically denied the leakage of fluid.

### 2.3 Prenatal US findings

**Figure 1:**
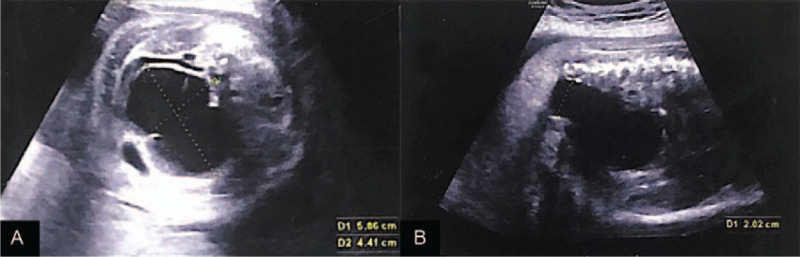
Prenatal US findings. (A) It showed a 5.9 × 4.4 cm cystic mass in the abdominal cavity. (B) It also indicated that the rectum was widened, and the diameter of the widest part was about 2.0 cm.

The US indicated a single fetus in cephalic presentation and a healthy placenta. Double apex diameter of about 8.1 cm, head circumference of 28.3 cm, the abdominal circumference of about 32.3 cm, the femoral length of about 5.5 cm, humerus length of approximately 5.1 cm, fetal heart rate of about 165 beats/min, and umbilical artery S/D value of about 2.3 were measured. The fetal head and neck were healthy. The size of the right kidney was about 5.5 × 2.4 cm, and 2 sets of collection systems could be seen in the right kidney. The lower collection system was separated by about 1.2 cm thick, while the upper collection system was separated by about 1.0 cm. The dilated ureter was visible, and the wide part of the ureter was about 0.44 cm. The size of the left kidney was about 4.2 × 2.4 cm, and the collection system was separated by about 1.1 cm. A 5.9 × 4.4 cm cystic mass was found in the abdominal cavity of the fetus (Fig. 1A), and another cystic mass was seen in the ventral side of this cystic mass, with a size of about 3.0 × 1.1 cm. The “target ring sign” of the fetal anus was not shown, and the fetal rectum was widened to approximately 2.0 cm (Fig. 1B). The lower margin of the placenta was about 3.3 cm from the cervical orifice, the amniotic fluid depth was about 6.6 cm, and the amniotic fluid index was 15.

### 2.4 Prenatal MRI characteristics

**Figure 2:**
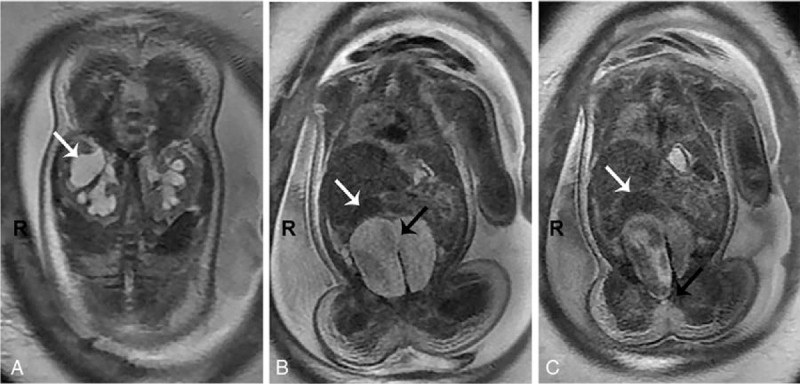
Prenatal MRI results. (A) The double collection system (white arrow) was found in the right kidney; (B) septum can be seen in the midline of the capsule (black arrow), but the upper part of the capsule is interconnected (white arrow); (C) the right cystic cavity passes down in the rectum and anal region (black arrow), and its upper end appears to be connected with the intestine (white arrow). MRI = magnetic resonance imaging.

Fetal MRI revealed dilatation and effusion of the bilateral kidney calices, kidney pelvis and ureter, low signal in the T1-weighted phase, the high signal in the T2 weighted phase, and a double-collecting system in the right kidney (Fig. 2A). An oval-shaped saclike sign of T2 hyperintensity was seen in the lower abdomen of the fetus. The septum was observed in the midline of the capsule, but the upper part of the capsule was interconnected (Fig. 2B). The right cystic cavity passed down in the rectum and anal region, and its upper end appeared to be connected with the intestine (Fig. 2C). The size and shape of the liver were normal, with no apparent abnormal signal. The size and shape of the spleen were normal and no abnormal signal was observed.

### 2.5 Treatment and clinical outcomes

**Figure 3:**
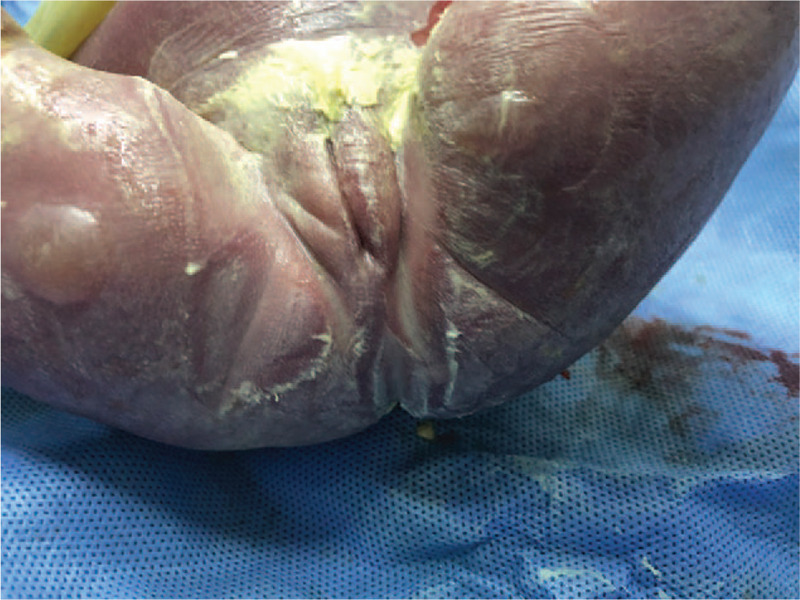
Clinical picture of the neonate CM. The newborn has no anus. CM = cloaca malformation.

After fetus CM was diagnosed by prenatal US and MRI, the amniotic cavity of pregnant women was injected with 100 mL of ethacridine lactate injection (Jiangsu Deseno Pharmaceutical Co., Ltd., China) under ultrasonic guidance. A total of 48 hours later, the gravida delivered a dead female neonate without anus (Fig. 3) through the vagina. The postpartum woman received symptomatic management, including prevention of infection and promotion of uterine contraction.

We also evaluated the general condition of the neonate, gaining the weight of 2280 g, the length of 39 cm, the head circumference of 26.3 cm, the abdomen circumference of 31 cm, the right thigh circumference of 17 cm, and the left thigh circumference of 18 cm. In addition, the abdomen was bulging and the palpation texture of bilateral thighs was relatively hard.

## 3 Discussion

CM is a quite rare deformity showing only one opening around the perineum, which is caused by the failure of cloacal division in the early embryonic development.^[13]^ CM is commonly found in female newborns.^[2,3,5,6,11,13,14,17]^ As far as we know, only 2 male neonates have been reported in the literature.^[4,8]^ Depending on the different stages of development stop, it will lead to various spectrum malformations, ranging from urogenital sinus to cloacal dysgenesis. To date, the prenatal diagnosis of female urogenital anomalies is still challenging due to the rarity of the condition. Therefore, we report a rare case of CM in which prenatal US and MRI were used to make a confident prenatal diagnosis.

### 3.1 The origin and spectrum of CM

Concerning the origin of CM, the cloaca develops into the lower rectum, anal canal, anus, bladder, urethra, and genitalia. It is a common channel in the early stage of embryo development. With the development of the embryo, it is gradually separated into three disconnected channels, including the urethra, vagina, and rectum.^[18]^ Williams Iv et al^[19]^ and Wheeler and Weaver^[20]^ identified hedgehog signaling, hereditary causes, or hormonal dysregulation as the risk factors of CM. These factors can affect embryonic development and eventually lead to the fusion of 2 or 3 channels in the vagina, urethra, and rectum.

Depending on the position of the opening around the perineum, CM can be further divided into the persistent cloaca and posterior cloaca.^[21]^ The former has a single shared channel located at the perineal site, while the latter has a single common pathway for the urethra, vagina, and rectum situated near the anus. In the present study, the newborn was classified as persistent cloaca due to a single shared channel was detected at the perineal site.

### 3.2 Ultrasound findings

US images can indicate different features because CM can be revealed in a broad spectrum of variations. A previous study^[22]^ suggested that 3 ultrasound findings can especially indicate CM, including intra-abdominal cystic mass, loop dilatation, and abnormal urinary tract.^[22]^ Winkler et al,^[8]^ reported US results of 6 newborns with CM, all suggesting cystic pelvic mass. Moreover, observation of 2 or 3 cysts increases specificity.^[10]^ In our case, 2 cystic masses can be observed in the lower abdominal cavity of the fetus. Another scholar believes that the extensive US, or even MRI, should be performed if an intraabdominal enlarged cystic mass, underdeveloped bladder, hydronephrosis, spinal deformity, or ambiguous genitals is found in fetus.^[23]^

Fetal ascites can be found occasionally in the US. Staboulidou et al,^[24]^ think it is fetal urine. This is because fetal urine can flow into the abdominal cavity through the fallopian tube in the case of complete outlet obstruction. In addition, peritoneal calcification can also be observed when the fluid reflux contains meconium, leading to meconium peritonitis.^[8]^

Hydronephrosis, as an isolated disease, is very common in prenatal ultrasound. In most cases, it disappears spontaneously after birth. Still, it may also be the result of a potential deformity. Levitt and Peña,^[25]^ reported that 90% of CM patients had abnormalities of the urinary system, among which bilateral hydronephrosis was the most common manifestation. In our study, the fetus presented bilateral hydronephrosis with right renal duplication, consistent with previous research.^[25]^ In addition, progressive vaginal enlargement is another US findings of CM patients since urinary outlet obstruction may cause urinary reflux into the vaginal cavity.^[7]^

According to previous reports,^[1,3,6,11,12,17]^ the frequency of suspicious signs on the prenatal US in CM patients ranged from high to low are abdominal cystic mass, bilateral hydronephrosis, oligohydramnios, hydronephrosis, ascites, and dilatation of the bowel. In the present case, the patient has the right fallopian tube dilatation, which may be associated with CM.

### 3.3 Magnetic resonance imaging

Concerning the rational fetal MRI diagnosis of CM, MRI shows the gastrointestinal tract clearly and provides reliable evidence for the assessment of the rectum, bladder, and vagina.^[16,26]^ MRI of CM patients usually shows an increase in the content of distal intestinal fluid caused by the intestinal and urinary tract communication.^[15]^ In general, the MRI T2 signals of the bladder and vagina are different, but the CM patients show similar signals of the 2 compartments. This may be because the genital tract and urinary tract are interconnected due to the developmental malformation, ultimately resulting in communication signals.^[14]^ In this study, we can clearly observe the positional relationship among intestine, vagina, and bladder with the aid of MRI. Therefore, MRI is beneficial to evaluating pelvic anatomy, rectal contents, and abnormalities when the prenatal US cannot make a definitive diagnosis.

Calcifications in MRI are regarded as a specific prenatal feature of communication between the distal bowel and urinary tract. It is because that the mixing of meconium and urine in the distal intestine urinary and the tract system causes the formation of intraluminal calcifications. This phenomenon was initially reported by Zaccara et al,^[27]^ as anorectal deformity with fistula. It may also occur in CM patients. However, such signs occur only occasionally and are not easily detected at an early stage.^[9]^ The MRI examination of our case does not show this sign either. In short, if fetal MRI shows calcification in the distal intestine or urinary tract, CM should be highly suspected by radiologists.

MRI findings of rectourinary fistula are also valuable in the diagnosis of prenatal CM, which can be found in female fetuses with CM and male newborns with anal atresia.^[28]^ Abnormal signals caused by the increased fluid content in the distal part of the intestine may be another evidence to suspect CM,^[15]^ and the meconium signal layering in the bladder of CM neonates has been reported by Calvo-Garcia et al.^[9]^

Megacystis can be detected in various conditions, including megacystis-microcolonintestinal hypoperistalsis syndrome, urethral atresia or stenosis, and posterior urethral valve resection syndrome,^[15,29]^ but rare in CM patients with approximately 1 in 1500 pregnancies.^[9]^ The occurrence of megacystis in CM patients may partly attribute to mechanical obstruction resulting from narrow common channels.^[30]^ According to previous studies, it is believed that megacystis usually suggests an adverse prognosis.^[31]^

### 3.4 The clinical treatment options of CM

The initial treatment and definitive reconstruction of CM are the predominantly clinical treatment options that are technically demanding. With appropriate management, the patients could achieve an excellent anatomical repair.^[7]^ Sharma and Gupta^[2]^ reported that the initial diversion surgery in CM was a transverse colostomy. Further treatment depends on the length of the common channel, which needs a comprehensive evaluation before considering pull through. Meanwhile, the goal of definitive management for CM is to optimize the function of urologic, gynecologic, and gastrointestinal systems, which need to separate each of the 3 structures to create a perineal opening and a catheterize urethra.^[7,32]^

To sum up, the combination of US and MRI can offer reliable evidence for diagnosing fetal CM in the third trimester and provide essential information to the pregnant women to determine clinic treatment options as early as possible.

## Author contributions

**Methodology:** Ge Huang, Chang-Jun Zheng, Guang-Yu Chu.

**Validation:** Chang-Jun Zheng.

**Writing – original draft:** Ge Huang, Shu-Yan Liu.

**Writing – review & editing:** Shu-Yan Liu.
